# The effect of blood flow restriction exercise on N-lactoylphenylalanine and appetite regulation in obese adults: a cross-design study

**DOI:** 10.3389/fendo.2023.1289574

**Published:** 2023-12-05

**Authors:** Shuoqi Li, Rong Guo, Juncheng Wang, Xinyu Zheng, Shuo Zhao, Zhiru Zhang, Wenbing Yu, Shiming Li, Peng Zheng

**Affiliations:** ^1^ School of Sports Science, Nantong University, Nantong, China; ^2^ School of Foreign Languages, Ludong University, Yantai, China; ^3^ Department of Physical Education, Ocean University of China, Qingdao, China

**Keywords:** moderate-intensity continuous exercise, blood flow restriction, obesity, appetite regulation, N-lactoylphenylalanine, ad libitum

## Abstract

**Background:**

N-lactoylphenylalanine (Lac-Phe) is a new form of “exerkines” closely related to lactate (La), which may be able to inhibit appetite. Blood flow restriction (BFR) can lead to local tissue hypoxia and increase lactate accumulation. Therefore, this study investigated the effects of combining Moderate-intensity Continuous Exercise (MICE) with BFR on Lac-Phe and appetite regulation in obese adults.

**Methods:**

This study employed the cross-design study and recruited 14 obese adults aged 18-24 years. The participants were randomly divided into three groups and performed several tests with specific experimental conditions: (1) M group (MICE without BFR, 60%VO_2max_, 200 kJ); (2) B group (MICE with BFR, 60%VO_2max_, 200 kJ); and (3) C group (control session without exercise). Participants were given a standardized meal 60 min before exercise and a ad libitum 60 min after exercise. In addition, blood and Visual Analogue Scale (VAS) were collected before, immediately after, and 1 hour after performing the exercise.

**Results:**

No significant difference in each index was detected before exercise. After exercise, the primary differential metabolites detected in the M and B groups were xanthine, La, succinate, Lac-Phe, citrate, urocanic acid, and myristic acid. Apart from that, the major enrichment pathways include the citrate cycle, alanine, aspartate, and glutamate metabolism. The enhanced Lac-Phe and La level in the B group was higher than M and C groups. Hunger of the B group immediately after exercise substantially differed from M group. The total ghrelin, glucagon-like peptide-1 and hunger in the B group 1 hour after exercise differed substantially from M group. The results of calorie intake showed no significant difference among the indexes in each group.

**Conclusions:**

In conclusion, this cross-design study demonstrated that the combined MICE and BFR exercise reduced the appetite of obese adults by promoting the secretion of Lac-Phe and ghrelin. However, the exercise did not considerably affect the subsequent ad libitum intake.

## Introduction

The prevalence of obesity is gradually rising in line with the continuous improvement of living standards (1). Obesity can easily trigger metabolic-related diseases, such as hypertension and diabetes, that are harmful to human health (2–4). In this regard, exercise is essential in alleviating obesity complications (5) and promotes negative energy balance by consuming more energy (6). Moreover, exercise has been reported to help individuals control hunger, satiety, and body weight by directly influencing the secretion level of certain appetite-related hormones in the blood circulation (7, 8).

A large number of previous studies have examined the reactions related to appetite during and after a single sustained aerobic exercise, with most of these studies conducted in physically active male (9–12). Overall, these studies indicate that subjective appetite is temporarily suppressed during exercise with an intensity greater than 60% of the maximum oxygen uptake. This phenomenon is called anorexia caused by exercise (13). Appetite perception usually returns to resting control values within 30 to 60 minutes after stopping exercise (13, 14), and they do not affect energy or constant nutrient intake on the day of exercise (15). However, the mechanism by which exercise affects appetite is currently unclear, and it is speculated that it may be related to the secretion of appetite hormones (7).

Apart from appetite-regulating hormones, an increasing number of metabolites have been shown to indicate metabolic spillovers and significantly mediate cross-talk between different organs and cells, thus providing favorable effects of physical activities (16, 17). N-lactoylphenylalanine (Lac-Phe) is a type of pseudo-dipeptide synthesized from lactate (La) and phenylalanine (Phe) that increases rapidly in the blood, specifically during and right after performing physical exercise (18). Recent studies have considered Lac-Phe as a new form of “exerkines” that can inhibit appetite in obese mice and reduce their weight and mass of adipose tissues (19, 20). In addition, the significant secretion of Lac-Phe during high-intensity exercise is closely linked to the La secretion level (21).

Blood Flow Restriction (BFR) exercise has received considerable interest over the past years as a new exercise intervention method (22). Generally, BFR limits the blood flow during exercise, elevates the mechanical pressure of working muscles, and causes local hypoxia/ischemia (23, 24). BFR exercise also generates higher lactic acid accumulation than non-BFR exercises (25). Despite that several research have demonstrated the reduction of fat mass over long-term BFR exercise (26, 27), the exact mechanism remains unclear, and the influence of BFR on regulating appetite is still ambiguous. Therefore, this study investigated the effects of combining moderate-intensity continuous exercise (MICE) with BFR on Lac-Phe and appetite regulation in obese adults.

## Methods

### Selection of participants

Participants were recruited from Ocean University of China via email, social networks, posters, and leaflets. The selection of participants in this study was based on the following criteria: obese adults aged 18-24 years, possess a Body Mass Index (BMI) of 30–39.9 kg/m^2^, were previously sedentary, and had not been prescribed any medication. In addition, those with respiratory and cardiovascular diseases, grade 3 obesity (BMI ≥ 40 kg/m^2^), diabetes, arterial hypertension, anemia, and cancer were excluded. The experimental procedures involving human subjects in this study follow the principles of the Declaration of Helsinki and were approved by the Ocean University of China research ethics committee (protocol No.:OUC-HM-2023-005). All participants agreed and filled out a written informed consent.

### Study design

**Figure 1:**
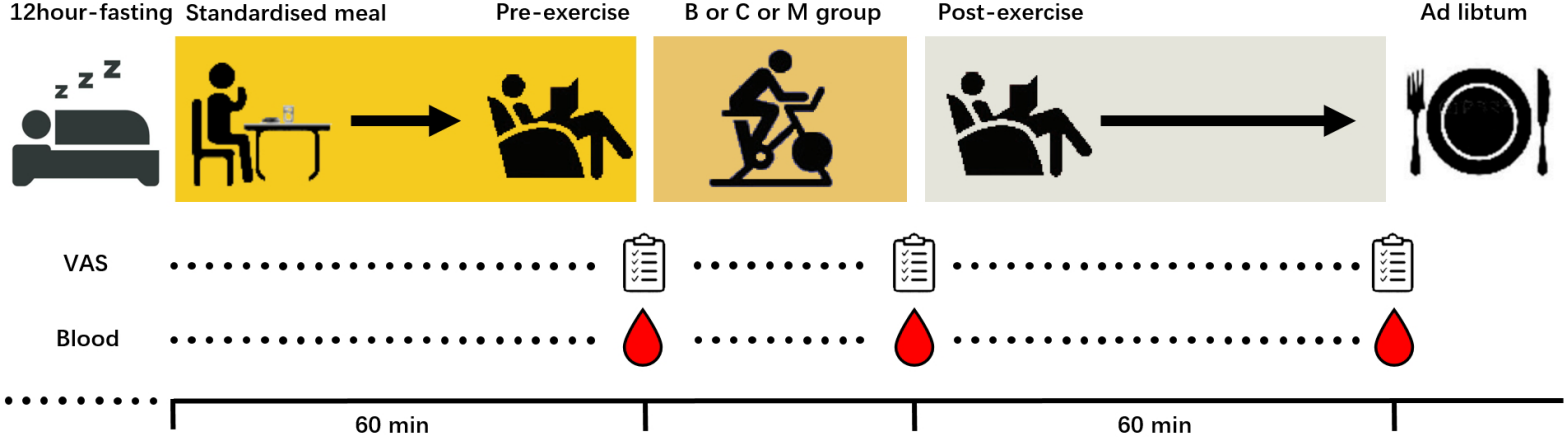
The flowchart of the experimental sessions.

This study employed the cross-design study. Before the experimental sessions, the participants were briefed on the test content and baseline measurement before performing the incremental test using an LC6 novo ergometer (Monark, Sweden) to determine the exercise intensity in the trial. **Figure 1** presents the flow diagram of the three experimental sessions. Prior to the trial, participants were required to fast for 12 hours in order to avoid the effect of diet at night on the baseline appetite. They were then instructed to enter the laboratory at 8 a.m. and undergo each session until the test was completed. In the follow-up session, the participants were randomly divided into three groups and performed several tests with specific experimental conditions: (1) M group (MICE without BFR, 60% VO_2max_, 200 kJ); (2) B group (MICE with BFR, 60%VO_2max_, 200 kJ); and (3) C group (control session without exercise). The seat height was maintained consistently during each exercise, and heart rate and Rating of Perceived Exertion (RPE) data were collected every 5 minutes during the exercise.

Participants were given a standardized meal 60 min before exercise (8 a.m) and a ad libitum 60 min after exercise. In addition, considering the changes pattern in lac-phe after exercise, blood and Visual Analogue Scale (VAS) were collected before, immediately after, and 1 hour after performing the exercise. The collected plasma was used to test appetite hormones and metabolomics. To minimize fluctuation, participants were instructed to register their food intake 24 hours before the first experimental session and eat the same food 24 hours before each follow-up session. Participants were reminded to avoid strenuous exercise, caffeine, and alcohol intake. In addition, the washout period was 7 days for males and 28 days for females to ensure all experimental sessions were conducted during the follicular phase of the menstrual cycle (Determined by luteinizing hormone test paper).

### Anthropometric and body fat percentage assessment

At baseline measurement, the weight of each participant (barefooted and wearing light clothes) was measured using an MI2 digital scale (Xiaomi, Beijing, China) with a maximum weight capacity of 100 kg and an accuracy of 50 g. Next, the participants’ height was measured using a fixed height ruler with an accuracy of 1 mm. Finally, the body fat percentage of each participant was calculated using dual-energy X-ray absorptiometry (Discovery Wi, Hologic Inc, USA).

### Maximum oxygen uptake test

The gradient cycling exercise protocol was employed to measure the VO_2max_, beginning from 60 W with a pedal frequency of 60 revolutions per minute (rpm). The power output was increased every 2 min at 40 and 20 W for males and females until they became exhausted. During the test period, the Heart Rate (HR) and VO_2_ were evaluated using an H12 Heart Rate monitor (Polar, Finland) and Quark-PFT gas metabolism analyzer (COSMED, Italy), respectively. Oxygen uptake is collected by breath by breath, and the average value is extracted every 30 seconds. Note that the VO_2max_ represents the highest mean value in 30 seconds.

### Exercise protocol

Participants performed different exercise protocols in each group. Participants in the M group were instructed to exercise on an electronically braked cycle ergometer (Monark, Sweden) under continuous movement at a working rate corresponding to 60% VO_2max_ and a pedal frequency of 60 rpm, with a total work of 200 kJ. Participants in the B group also performed the same exercise protocol as those in the M group. However, they were fitted with an inflatable cuff with 60% Limb Occlusive Pressure (LOP) at the proximal end of the thigh to limit blood flow. On the contrary, participants in the C group only sat still for the same period and did not perform any exercise.

During the exercise, the HR and RPE of each participant in the M and B groups were recorded every 5 min. The work rate corresponding to 60% VO_2max_ intensity was also determined based on the linear regression between the steady-state VO_2_ and power output established in the maximum oxygen uptake test before the test session. Subsequently, the total exercise time of the two exercise groups to complete 200 kJ (200 kJ work done/exercise work rate) was calculated.

### Limb occlusive pressure

Prior to the LOP measurement, all participants were fitted with an around 7 cm wide BFR cuff (Occlusion Cuff, Belfast, Northern Ireland). After laying on a bed, an ultrasonic Edan pocket Doppler probe (Northampton, United Kingdom) was put onto the participant’s tibial artery to measure auscultation pulses on the leg arterial occlusive pressure (mmHg) (28). Then, the cuff was placed at the thigh groin region before being slowly inflated. The LOP is defined as when the cuff pressure is interrupted by an auscultation pulse.

### Standardized meal

After 12 hours of fasting, a standardized liquid meal (UP&GO™, Sanitarium, Australia) was provided to each participant 60 min before the exercise. According to the manufacturer, each 100 g of the commercial liquid meal powder contains 328 kcal (carbohydrates = 61.4%, proteins = 20.8%, and lipids = 17.8%), providing each participant 4.5 kcal × body weight (kg). The content of the commercial meal complies with the recommended nutritional intake for pre-exercise calorie amount and macronutrient distribution (29).

### Ad libitum

The ad libitum program in this study follows the method used by Matos et al. (30). The ad libitum was served in a reserved room as a “buffet” 1 hour after the experimental sessions, and the participants could intake food “until they felt comfortably satisfied” in the room. The buffet contains an assortment of foods, including apples, bananas, boiled eggs, chocolate, fruit juice, jam, natural yoghurt, potato chips, and toast (**Table S6**). Each type of food was weighed before and after being served, and the dietary intake was evaluated using Dietwin Professional plus^®^ version 2.0 food analysis software (Porto Alegre, RS, Brazil).

### Appetite perception

Referring to previous testing methods (30), subjective appetite perception was conducted in each group at three time intervals: pre-exercise, post-exercise, and post-1-hour exercise. Subsequently, a visual analogue scale was utilized to record the appetite perception measurements based on four aspects: fullness, hunger, Perspective of Food Consumption (PFC), and satiety. The scale, which is an effective and reproducible tool (31, 32), comprises four 100 mm horizontal lines indicating the appetite perception level, with full perception on the left side of the scale and minimal or no perception on the right extremity. Before collecting data, participants were already familiar with the content of the scale, with the text marked “not at all” on the far right and “extremely” on the far left. At the above three time points, participants were instructed to indicate their perception level by marking a vertical line on the horizontal scale.

### Blood collection and enzyme-linked immunosorbent assay

Participants were seated, and approximately 5 mL of venous blood was drawn from the anterior cubital vein by venipuncture. The blood sample was collected in a heparin anticoagulant tube and centrifuged at 2000 g for 10 min at 4 °C. Then, the upper plasma layer was extracted and stored in a refrigerator at -80 °C. The total ghrelin (Millipore, Burlington, USA; % coefficient of variation (CV): 6.2%) and peptide YY (PYY) (Millipore, Burlington, USA; %CV: 4.8%) in the extracted sample were determined using radio-immunoassays, while glucagon-like peptide-1 (GLP-1) (Cusabio; %CV: 5.7%) and high sensitivity interleukin-6 (IL-6) (Multi science, Hangzhou, China; %CV: 6.7%) was measured using ELISA.

### Metabolite sample preparation

The samples were first lyophilized using a lyophilizer (Toffon, Shanghai, China). Then, a mix mill (Retsch, Haan, Germany) equipped with zirconia beads was used to ground the sample into powder form at 35 Hz for 1 min. Approximately 100 mg of the powder sample was then mixed with 70% methanol to achieve 0.1 g/mL of aqueous solution. The metabolites were extracted by ultrasonicating the aqueous sample mixture at 40 Hz for 10 min, followed by centrifugation and filtering.

### Untargeted metabolomics analysis

The collected plasma samples before and after exercise were subjected to untargeted metabolomics analysis using High-performance Liquid Chromatography. In addition, each sample was analyzed using Q Exactive Focus Orbitrap Liquid Chromatography-Mass Spectrometry/Mass Spectrometry (LC-MS/MS) (Thermo Scientific, Waltham, USA) operated in the full-scan mode. The electrospray ionization source parameters were conducted as follows: nebulising gas flow of 3 L/min; the heating gas flow of 10 L/min; interface temperature of 500 °C; DL temperature of 250°C; heat block temperature of 400°C; and drying gas flow of 10 L/min. The obtained raw data was analyzed using Compound Discoverer 3.3 software.

In the Compound Discover 3.3 software, high-quality (S/N > 10) metabolic signals from the MS2 spectral information were first compared with the database, and the metabolic signals were annotated in batches. Metabolic signals that were unmatched by the information in the database were identified by querying the MS2 spectral data from past literature or searching in other online databases, such as METLIN and MassBank. Compounds with standards were identified by comparing the Retention Time (RT), accurate m/z values, and fragmentation patterns with those obtained by injecting the standards under similar conditions.

### Targeted metabolomics analysis

Targeted metabolomics analysis was performed to determine the Lac-Phe, La, and Phe levels in the collected plasma samples using a Vanquish Ultra High-performance Liquid Chromatography-Mass Spectrometry coupled with a Q Exactive operated in negative ion mode, as described by Hoene et al. (21) with minor adjustments. Approximately 50 µL of the plasma sample was mixed with 250 µL of methanol and vortexed for 30 seconds before being centrifuged at 16,000 g for 20 min at 4 °C. After aliquots of 200 µL supernatant were vacuum-dried, the dried samples were resuspended in 50 µL of 25% ACN/water mixture.

An ACQUITY UPLC HSS T3 column (pore size = 1.8 µm, length = 2.1 × 100 mm) (Waters, Milford, USA) was equipped to carry out the separation. The mobile phases comprised (A) 6.5 mM ammonium bicarbonate ((NH_4_)HCO_3_) in water and (B) 6.5 mM (NH_4_)HCO_3_ in 95% methanol/water mixture. At a flow rate of 0.35 mL/min and a column temperature of 50 °C, the elution was initiated as follows: 2%B for 1 min, linearly changed to 100%B within 20 min, and reverted to 2%B before equilibrating for 2.9 min. The Q Exactive was operated in full-scan mode at 140,000 resolution with a mass scan range of 70–1050 m/z.

The spray voltage was operated at 3.00 kV. Nitrogen sheath gas and nitrogen auxiliary gas were fixed at a flow rate of 45 and 10 AU, respectively, while capillary and aux gas heater temperatures were set at 300 °C and 350 °C, respectively. Parallel reaction monitoring was employed to achieve high-resolution of the Lac-Phe MS/MS spectra (m/z = 236.093) at 17,500 resolution and collision energy of 30 eV. The signal intensities were normalized by adding 0.8 µg/mL of internal standard d5-Phe (615870, Merck, Germany) in the extraction solvent.

### Randomization and blinding

An online randomization tool (randomizer.org) was applied to generate the randomized sequences. After the generated sequences were placed and sealed in opaque envelopes. The envelopes were delivered to each participant before their first experimental session. Participants were informed of the type of study session (B, M, or C group session) upon arrival for each study session.

### Data analysis

MetaboAnalyst 5.0 (33) is a practical multi-tool to perform and interpret metabolomics analysis (www.metaboanalyst.ca) and was used to process the obtained dataset. When required to use each module, data was uploaded in the form of a complete dataset containing the chemical name or as a restricted list of Human Metabolome Database (HMDB) IDs. Data filtering was utilized based on the Interquartile Range (IQR) specifically for untargeted metabolomics datasets. Abundance data was uploaded in the form of a csv. file in unpaired rows, and sample normalization was obtained using log transformation and Pareto scaling (mean-centered divided by the square root of the standard deviation of each variable). Besides, Partial Least Squares Discriminant Analysis (PLS-DA) was employed to predict the model’s reliability. A Fold Change (FC) ≥1.5 or ≤0.67 and P value < 0.05 were applied to screen distinctly expressed metabolites. The KEGG database was then searched to enrich the metabolic pathways.

G-Power software version 3.1.9.6 (University of Trier, Germany) was utilized to calculate the sample size. Additionally, the normal distribution of the variables was assessed using the Shapiro-Wilk normality test, while the differences in appetite scale, appetite hormone, and targeted metabolomics were evaluated across the varying time intervals and different participant groups using the two-way repeated-measure Analysis of Variance (ANOVA). For significant interactions, *Post-hoc* ANOVA was applied based on the Bonferroni test to identify the simple main effects. Paired sample t-test was used to analyze the calorie intake of the ad libitum in each group. Results for statistical significance are indicated with a P < 0.05. Data were expressed as the mean ± standard deviation (SD).

## Results

**Table 1** displayed the physical features of 14 participants who completed the three sessions without adverse events, while **Table 2** lists the exercise protocol of the M and B groups. Accordingly, the average HR and RPE of the B group were significantly greater than the M group (P<0.05). The power in the *post hoc* test is 0.98.

**Table 1:** Physical characteristics of participants (n=14).

Item	Value
Age(y)	20.6±1.5
Gender(Female:Male)	7:7
Weight(kg)	88.7±15.1
Height(cm)	168.5±12.7
BMI(kg/m^2^)	31.1±2.0
BF(%)	38.8±3.7
VO_2max_(ml/min/kg)	33.1±4.5

BMI, Body mass index; BF, Body fat percentage; VO_2max_, Maximal oxygen uptake.

**Table 2:** Details of exercise protocol (n=14).

Item	M group	B group
HR(beat/min)	133.4±3.6	145.6±4.4^*^
RPE	12.7±0.9	14.4±0.8^*^
Work rate(W)	71.6±17.9	71.6±17.9
Work done(kJ)	200	200
Duration(min)	49.5±13.2	49.5±13.2
LOP(mmHg)		202.9±11.6
60%LOP(mmHg)		121.8±7.0

HR, Heart rate; RPE, Rating of perceived exertion; LOP, Limb occlusive pressure.*, There is a significant difference compared to M group. The statistical method uses paired sample t-tests.

**Figure 2:**
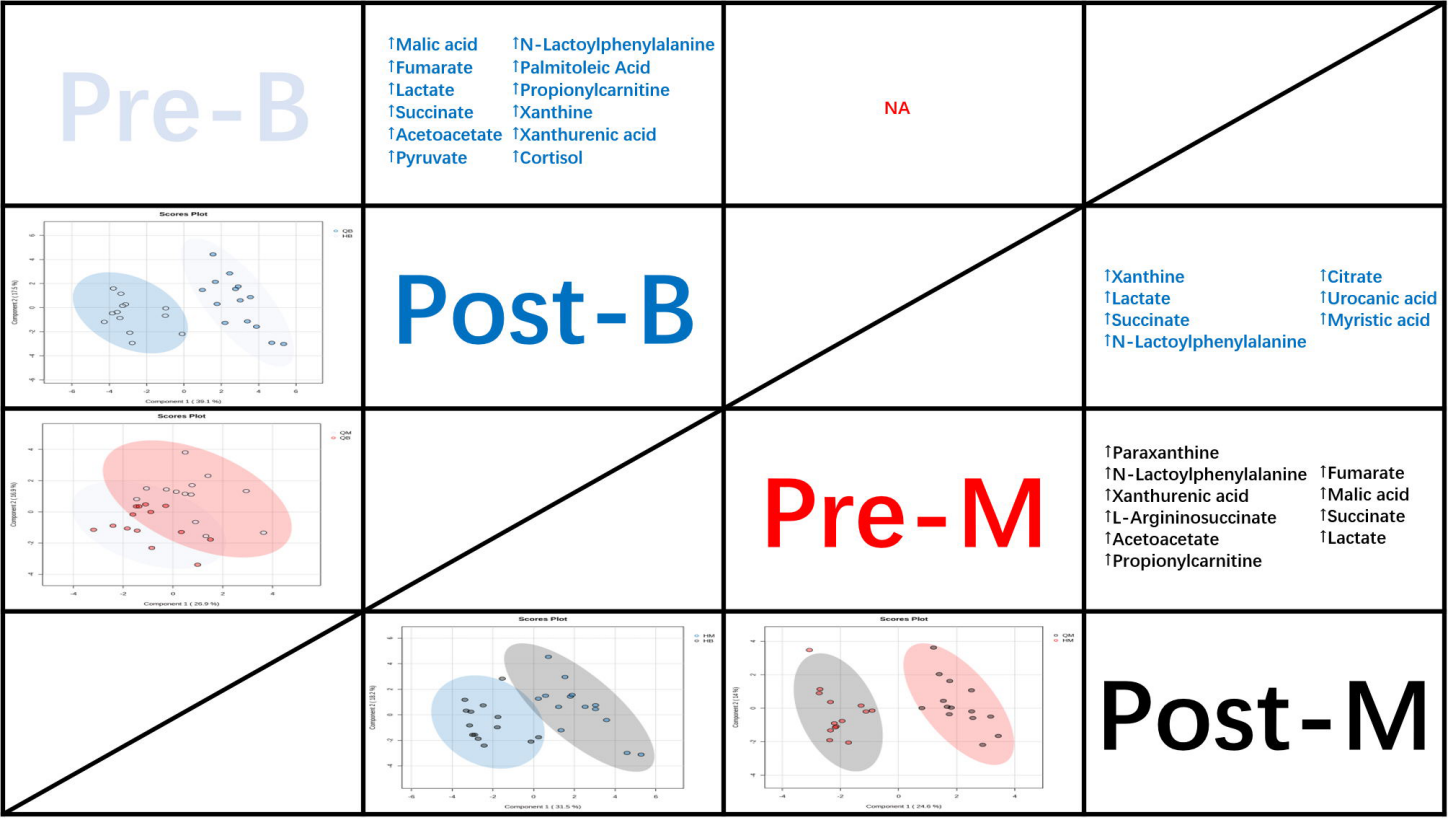
PLS-DA score plots and the trend of major metabolite levels (n=14). Note: The panels under the diagonal display PLS-DA score plots demonstrate the different metabolic profiles between study interventions (Pre-B, cyan; Post-B, blue; Pre-M, red; and Post-M, black).

**Figure 3:**
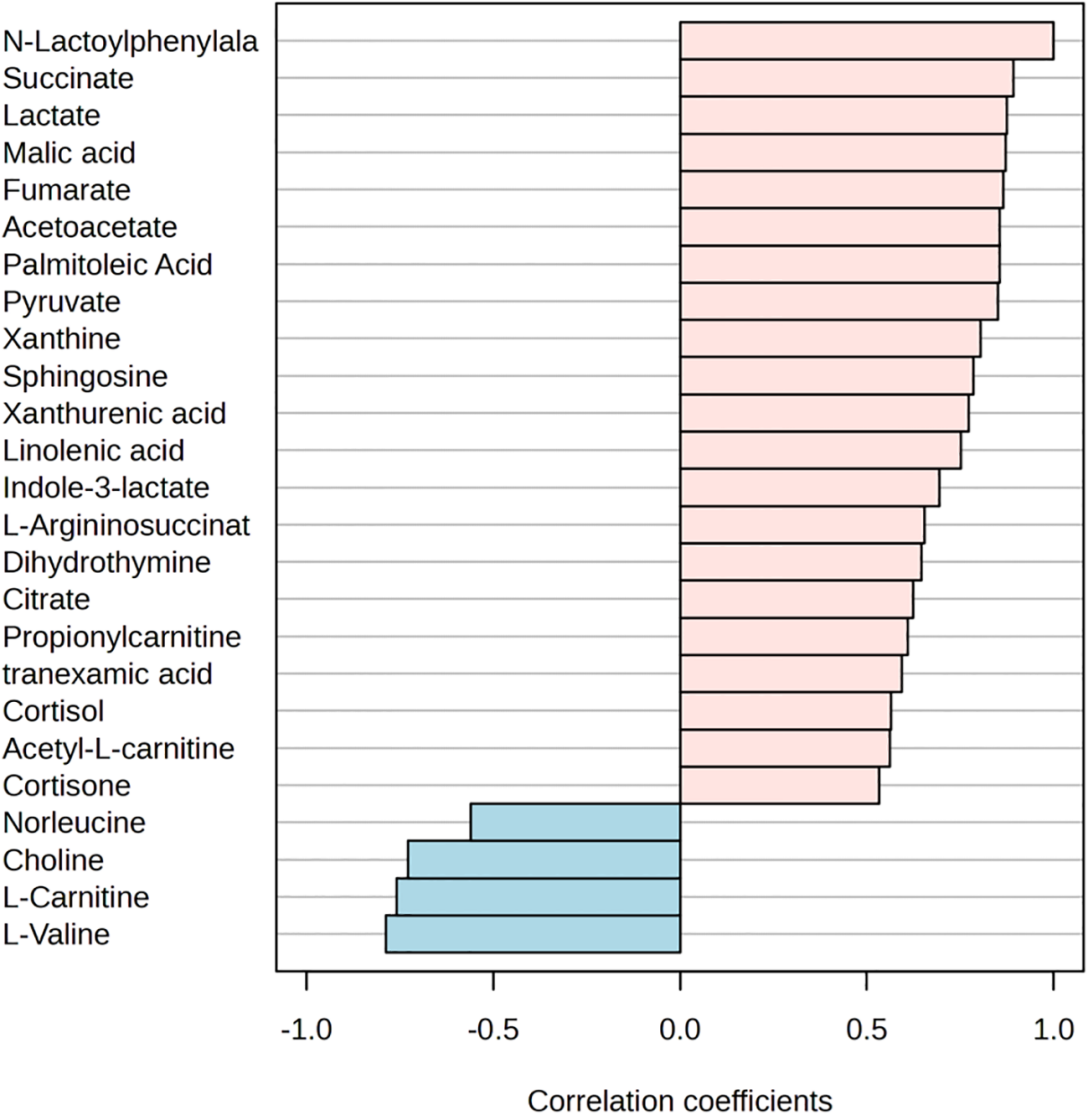
Top 25 metabolites correlated with Lac-Phe (n=14).

The PLS-DA model score plots in **Figure 2** indicate the trend of major metabolite levels between the M and B groups before and after exercise. Except for Pre-B and Pre-M, all other groups exhibited high distinguishability. After exercise, the primary differential metabolites detected in the M and B groups were xanthine, La, succinate, Lac-Phe, citrate, urocanic acid, and myristic acid. Apart from that, the major enrichment pathways include the citrate cycle, alanine, aspartate, and glutamate metabolism. **Figure 3** shows the top 25 metabolites linked to Lac-Phe, and the correlation diagrams of other metabolites in the citrate cycle are provided in the appendix.

**Figure 4:**
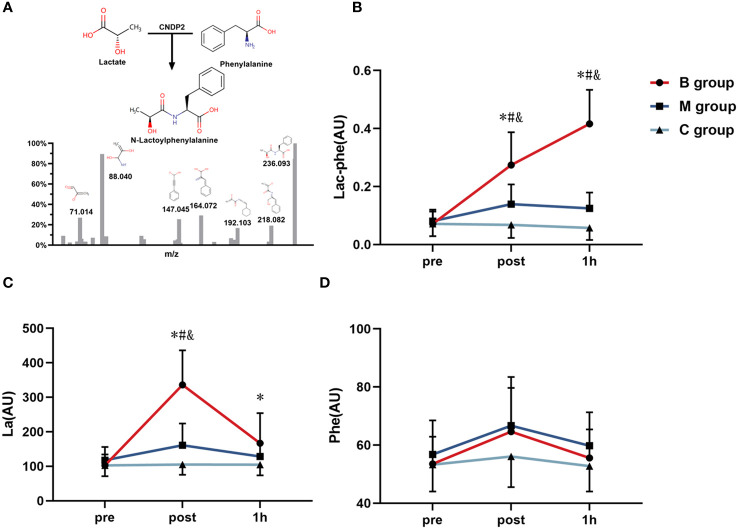
Detection of **(A)** Lac-Phe and the changing level of **(B)** Lac-Phe, **(C)** La, and **(D)** Phe after exercise in each group (n=14). Lac-Phe, N-lactoylphenylalanine; La, Lactate; Phe, Phenylalanine; * = significant difference between B group and C group; ^#^ = significant difference between M group and C group; ^&^ = significant difference between B group and M group. The statistical method uses one-way analysis of variance.

The untargeted metabolomics LC-MS/MS analysis identified 103 metabolites, and the presence of Lac-Phe after exercise was confirmed. The analysis also detected three metabolites directly related to Lac-Phe, including Lac-Phe, La, and Phe, and was used in subsequent analyses. As shown in **Figure 4A**, the LC-MS/MS analysis identified Lac-Phe in all samples and were quantified. Comparatively, **Figure 4B** shows the level of Lac-Phe after exercise. The results indicated an insignificant difference in Lac-Phe levels between each group before exercise. However, the Lac-Phe level immediately and 1 hour after exercise in the B group was substantially increasing than in the M and C groups (P<0.05). The repeated ANOVA measurement results also showed significant interactions between the B, M, and C groups, where the enhanced Lac-Phe level in the B group was higher than M and C groups (P<0.05).

Furthermore, **Figure 4C** depicts the La level after exercise. The results imply an insignificant difference in La levels among the three groups before exercise. Nevertheless, the La level immediately after exercise in the B group was noticeably increasing than that of the M group and C group (P<0.05), and the La level 1 hour after exercise in the B group was substantially increasing than that of C group (P<0.05). Besides, the repeated ANOVA measurement results showed a significant interaction effect in the B group and M group compared to the C group (P<0.05), and the improved La level in the B group was significantly higher than that of the M and C groups (P<0.05). On the contrary, the Phe level after exercise in **Figure 4D** indicates no significant difference among the groups before exercise, immediately after exercise, and 1 hour after exercise.

**Figure 5:**
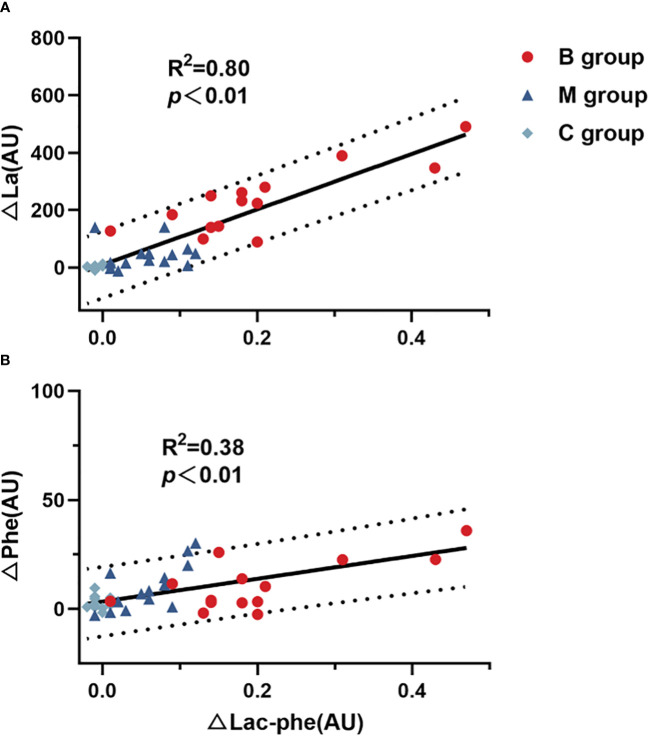
Correlation of difference in Lac-Phe with **(A)** La and **(B)** Phe (n=14). Lac-Phe, N-lactoylphenylalanine; La, Lactate; Phe, Phenylalanine. Pearson test was used for correlation analysis.

**Figure 5** presents the correlation of difference in Lac-Phe with La (**Figure 5A**) and Phe (**Figure 5B**) before and after exercise. The findings highlighted the significant correlation between Lac-Phe and La and Phe (P<0.01), with a strong association between Lac-Phe and La (R^2 =^ 0.80).

**Figure 6:**
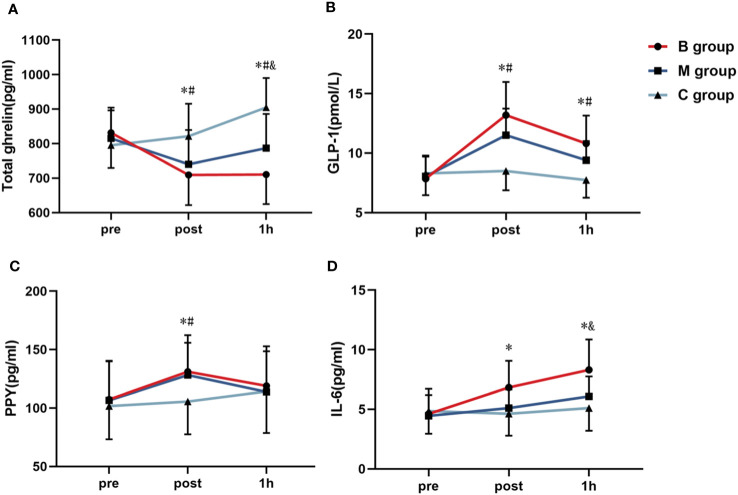
Line chart of the **(A)** total ghrelin, **(B)** GLP-1, **(C)** PPY, and **(D)** IL-6 before and after exercise (n=14). PPY, Peptide YY; IL-6, Interleukin-6; GLP-1, Glucagon-like peptide-1. * = significant difference between the B group and C group; ^#^ = significant difference between the M group and C group; ^&^ = significant difference between the B group and M group. The statistical method uses one-way analysis of variance.

**Figures 6A–C** illustrate the line chart of the total ghrelin, GLP-1, and PPY before and after exercise. No significant difference in each index was detected before exercise. However, the indexes of the B and M groups immediately after exercise substantially differed from C group (P<0.05). Interestingly, the total ghrelin and GLP-1 in the B and M groups 1 hour after exercise differed substantially from C group (P<0.05). Note that the total ghrelin in the B group was substantially decreased than M group (P<0.05). **Figure 6D** shows the IL-6 level before and after exercise. The level of IL-6 in the B group immediately after exercise was significantly higher than in the C group. The IL-6 level in the B group was also significantly higher than in the M and C groups 1 hour after exercise.

**Figure 7:**
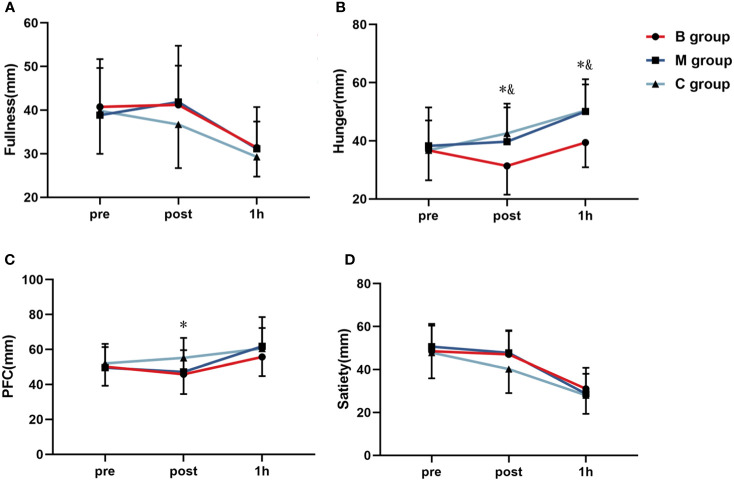
Line chart of **(A)** fullness, **(B)** hunger, **(C)** PFC, and **(D)** satiety before and after exercise (n=14). PFC, Perspective of Food Consumption. * = significant difference between B group and C group; ^&^ = significant difference between B group and M group. The statistical method uses one-way analysis of variance.

**Figure 7** shows the four VAS appetite subscales before and after exercise. No significant difference in each index was detected before exercise. In contrast, the hunger level immediately after exercise and 1 hour after exercise was substantially lower in the B group than in the M and C groups. Similarly, the PFC level immediately after exercise was significantly lower in the B group than in the C group.

**Figure 8:**
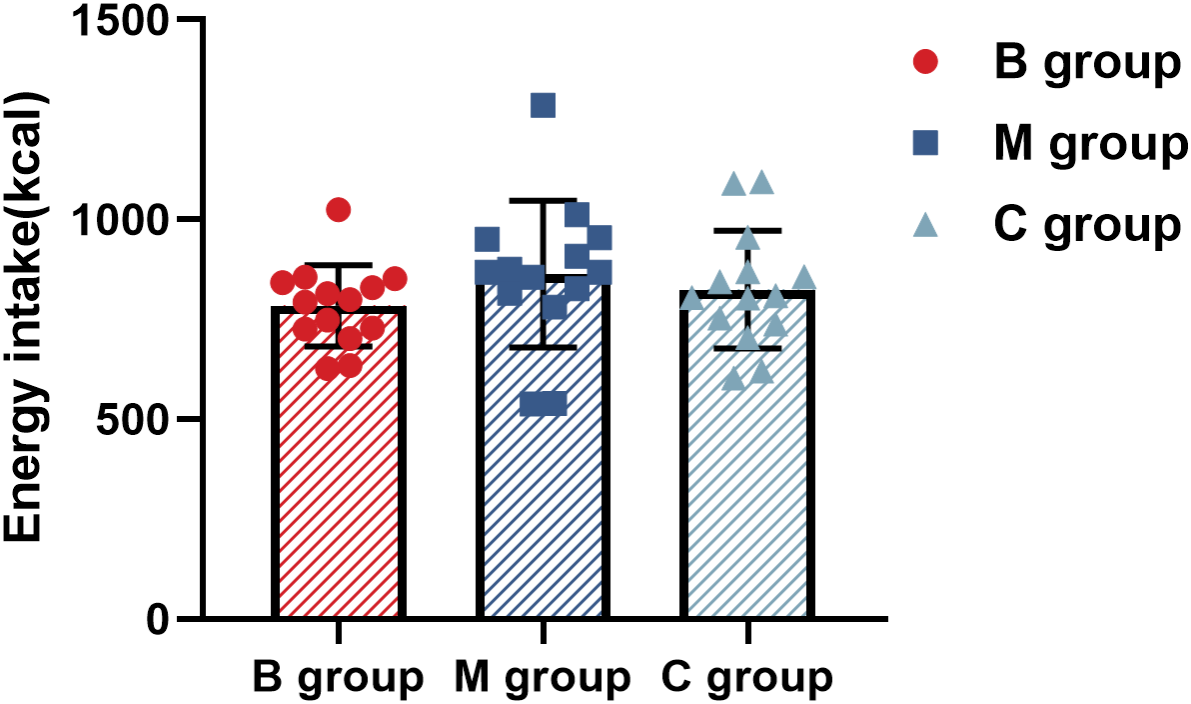
Calorie intake in ad libitum after exercise (n=14). The statistical method uses one-way analysis of variance.

**Figure 8** and **Table 3** present the macronutrients and calorie intake in the ad libitum after exercise, respectively. The results showed no significant difference among the indexes in each group.

**Table 3:** Macronutrients in ad libitum meal after exercise (n=14).

Items	B group	M group	C group
Carbohydrate(g)	104.29±19.19	118.95±30.32	110.99±26.48
Protein(g)	23.16±7.24	21.41±7.14	25.39±9.75
Lipids(g)	30.44±6.85	33.45±12.85	31.03±6.31

## Discussion

This study explored the acute effect of BFR exercise on appetite regulation. The results showed that the combined MICE with BFR could significantly reduce the subjective appetite perception after exercise compared to MICE without BFR but recorded no impact on the calorie intake of the ad libitum after exercise. The combined MICE with BFR also significantly increased the secretion of La and Lac-Phe and inhibited the secretion of appetite-related hormones. Note that BFR exercise could reduce subjective appetite perception by promoting the secretion of Lac-Phe.

The exercise program employed in this study was based on the previous MICE protocol specifically for obese individuals (34), which was proven to effectively enhance the body composition and reduce the body fat mass of obese women. The average HR of MICE was approximately 67.04% HR_max_, which fulfilled the definition of moderate-intensity. Moreover, the HR of MICE with BFR was substantially greater than that of MICE without BFR, consistent with past studies (5, 35).

Recently, Li et al. (19) recognized Lac-Phe as a new metabolite related to appetite regulation and showed that the concentration of Lac-Phe surged after performing the exercise. Following exogenous Lac-Phe injection in obese mice, their weekly calorie intake, body weight, and body fat were reduced without major changes in physical activities. In another study, the reduced visceral fat in obese individuals after long-term exercise intervention was predicted based on the increase in Lac-Phe level during acute exercise (21). However, a conclusive finding on the reduction of calorie intake could not be established since the type of diet consumed was not recorded in the study.

According to the present study, participants in the B group (MICE with BFR) recorded a higher Lac-Phe level immediately after exercise and 1 hour after exercise than participants in the M group (MICE without BFR). The outcome could be attributed to BFR preventing venous blood reflux and increased metabolic pressure during exercise (36). As a result, a higher accumulation of La after BFR exercise was recorded. In addition, the delay of the peak time of plasma Lac-Phe after exercise could be due to the need to synthesize Lac-Phe from La and Phe under the catalytic action of carnosine dipeptidase 2 (CNDP2) (19). A previous study by Li et al. (19) also revealed that the peak generation of plasma Lac-Phe occurred 30–60 min after exercise, which agrees with this study’s findings.

At the physiological and cellular level, human calorie intake and appetite are controlled by the neuro-endocrine system (37). Hormones secreted by the gastrointestinal tract mediate hunger and satiety to regulate appetite and calorie intake (38). So far, ghrelin is the sole orexigenic peripheral peptide known to be predominantly secreted by the gastric oxyntic cells and endocrine glands of the gastric mucosa (39). Ghrelin has three distinct forms: total, acylated, and des-acylated (40).

Based on the assessment of three appetite-related hormones (Lac-Phe, La, and Phe) in this study, it was revealed that only ghrelin showed different levels between the B and M groups. Ghrelin also showed a significant linear correlation with Lac-Phe (R^2 =^ 0.29, P < 0.01), which could be due to the mediation of La. Ghrelin-releasing gastric cells are highly enriched with G-protein-coupled receptor 81 (GPR81) (M.S. 41), where La acts as a ligand. Thus, La stimulation of the GPR81 dependently inhibits total ghrelin secretion in gastric mucosal cells (Maja S. 42). It should be highlighted that the secretion of total ghrelin in the present study was inhibited as a result of La treatment, implying that the La-mediated GPR81 signaling attenuates both the synthesis and activation of total ghrelin within these cells (42, 43).

The role of La in exercise-induced appetite inhibition with sodium bicarbonate (NaHCO_3_) supplement was explored in a cross-over trial research (7). A total of 11 healthy adult men performed high-intensity intermittent exercise twice with 90% HR_max_. Under the same exercise mode, the La level in blood samples of the experimental group was higher after exercise, which led to higher ghrelin levels, thus supporting the conclusion of this study. Therefore, BFR exercise may inhibit ghrelin production through La. Despite the secondary results assuming that BFR exercise could also promote GLP-1 secretion through IL-6 (15), the results were inconsistent with the previous hypothesis due to the irregular movement form or crowd. In addition, no differences in PYY between Group B and Group M were observed in this study, which may be due to the fact that PYY secretion is not related to lactate. In a previous study (15), the effects of different intensities of intermittent exercise on three appetite hormones were evaluated, and the correlation results showed that there was no significant correlation between PYY secretion after exercise and La and IL-6, which to some extent supports the results of this study.

While the results of this study demonstrated the impact of the combined MICE with BFR exercise on subjective appetite, total ghrelin, and Lac-Phe to a certain extent, the effect on the subsequent ad libitum intake was negligible. Similar inconsistent trends among overweight and obese subjects have also been reported in previous studies (30, 44). The acute change may not be sufficient to influence the clinical response since the desire and eating behavior are not entirely regulated by hormonal changes (45). Subjective appetite perception may not be a relevant parameter to predict calorie intake level, although it reflects the eating latency (46). Hence, any observed relationship with appetite perception may not necessarily represent the calorie intake in daily diet.

In addition, Lac-Phe had no impact on the calorie intake, probably due to the prolonged effect of Lac-Phe in mediating the eating behavior, although the observation time in this study was relatively short (60 min). In comparison, Li et al. (19) reported that a single exogenous Lac-Phe injection may have a long-term effect on changing the signal pathway of eating behavior in mice by up to 12 hours. Another study involving 17 overweight men who performed exercise with different intensities showed that a more intense exercise led to a higher La level after exercise, consequently reducing food intake on the following day (47). In short, BFR exercise coupled with MICE can enhance Lac-Phe and ghrelin secretion and reduce subjective appetite perception after exercise but has no impact on the subsequent ad libitum intake.

The procedure and outcome of the present study exhibit several limitations. Firstly, the sample size of this study only involved obese adults, which makes the results insignificant for other populations. Secondly, the impact of subsequent days and long-term BFR exercise on dietary calorie intake was not evaluated before performing this single acute study. Since the role of untargeted metabolomics analysis is only to screen and determine metabolites linked to Lac-Phe, it was not undetected 1 hour after exercise. Finally, this study emphasized only the effect of Lac-Phe induced by BFR exercise on appetite regulation and disregarded the influence of different exercise intensities and the number of exercise duration on Lac-Phe. Therefore, it is recommended that future studies employ broader and more diverse populations, perform randomized controlled trials to assess the effect of BFR exercise on dietary calorie intake, and investigate the mechanism of Lac-Phe in regulating appetite.

## Conclusion

In conclusion, this cross-design study demonstrated that the combined MICE and BFR exercise reduced the appetite of obese adults by promoting the secretion of Lac-Phe and ghrelin. However, the exercise did not considerably affect the subsequent ad libitum intake.

## Data availability statement

The raw data supporting the conclusions of this article will be made available by the authors, without undue reservation.

## Ethics statement

The studies involving humans were approved by The Ocean University of China research ethics committee (No.:OUC-HM-2023-005). The studies were conducted in accordance with the local legislation and institutional requirements. The participants provided their written informed consent to participate in this study.

## Author contributions

SQL: Conceptualization, Data curation, Formal analysis, Writing – original draft. RG: Data curation, Formal analysis, Writing – original draft. JW: Investigation, Methodology, Software, Writing – original draft. XZ: Project administration, Validation, Writing – review & editing. SZ: Investigation, Methodology, Writing – review & editing. ZZ: Resources, Visualization, Writing – review & editing. WY: Project administration, Validation, Writing – review & editing. SML: Conceptualization, Funding acquisition, Project administration, Resources, Supervision, Writing – review & editing. PZ: Conceptualization, Funding acquisition, Project administration, Resources, Supervision, Writing – review & editing.

## Supplementary material

Supplementary Figure 1 Volcano plot **(A)**, Heatmap **(B)**, variable importance in projection **(C)**, and enrichment analysis bubble map **(D)** of metabolites before and after exercise in B group (n=14).

Supplementary Figure 2 Volcano plot **(A)**, Heatmap **(B)**, variable importance in projection **(C)**, and enrichment analysis bubble map **(D)** of metabolites before and after exercise in M group (n=14).

Supplementary Figure 3 Volcano plot **(A)**, Heatmap **(B)**, variable importance in projection **(C)**, and enrichment analysis bubble map **(D)** of metabolites after exercise between B and M group (n=14).

Supplementary Figure 4 Correlation diagram of intermediate metabolites in the citric acid cycle (n=14).

Supplementary Figure 5 Correlation of N-Lactoylphenylalanine with total ghrelin **(A)** and hunger **(B)** (n=14). Pearson test was used for correlation analysis.

Supplementary Table 6 Food Items Provided in the Ad Libitum Buffet Meal.
