# Supplementary figures and images for: The effect of blood flow restriction exercise on N-lactoylphenylalanine and appetite regulation in obese adults: a cross-design study
Source: Front Endocrinol (Lausanne). 2023 Dec 5;14:1289574. doi: 10.3389/fendo.2023.1289574 (PMC10728722; doi:10.3389/fendo.2023.1289574)

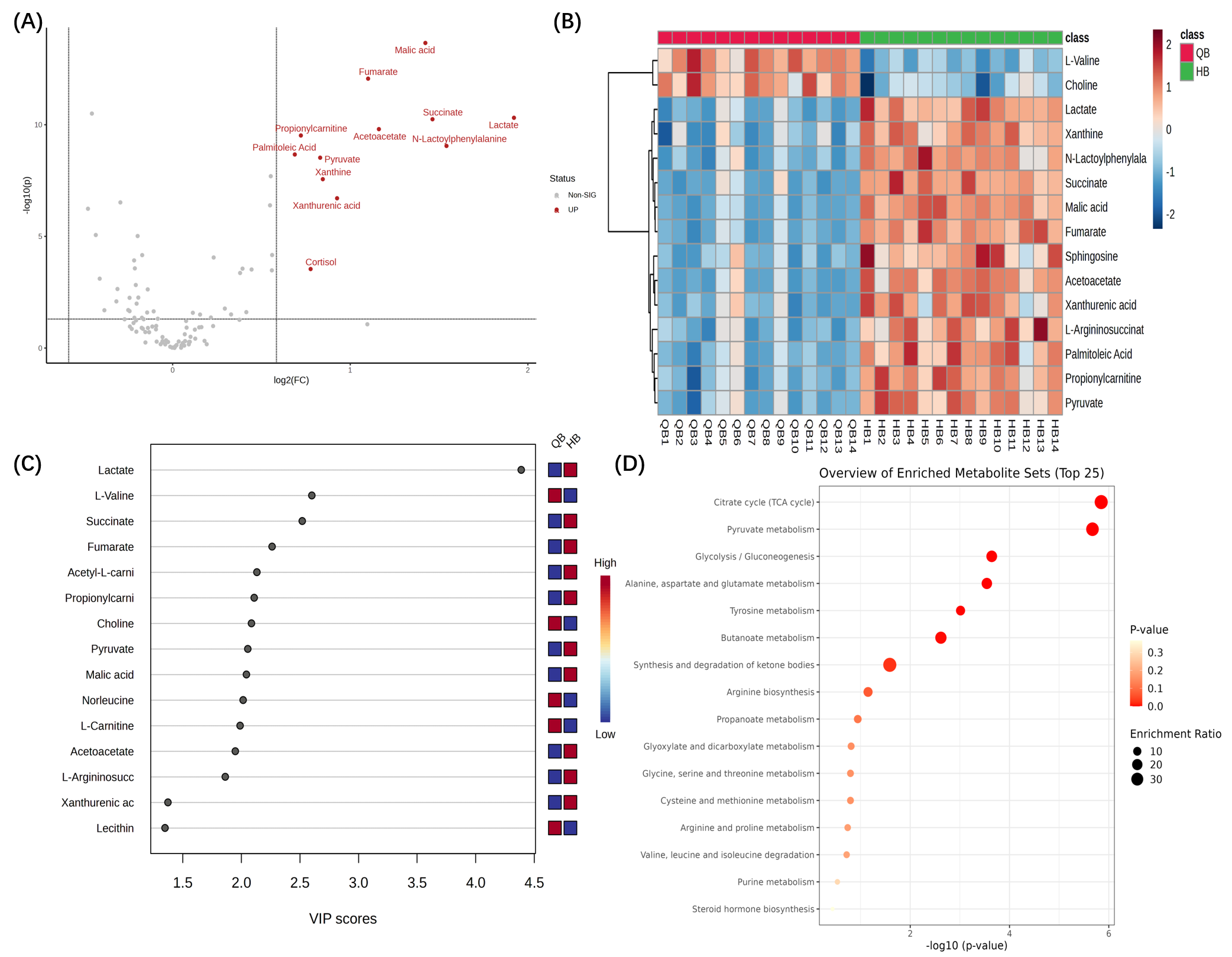

Supplement: Supplementary Figure 1 — Volcano plot (A), Heatmap (B), variable importance in projection (C), and enrichment analysis bubble map (D) of metabolites before and after exercise in B group (n=14). [file Image_1.tif]

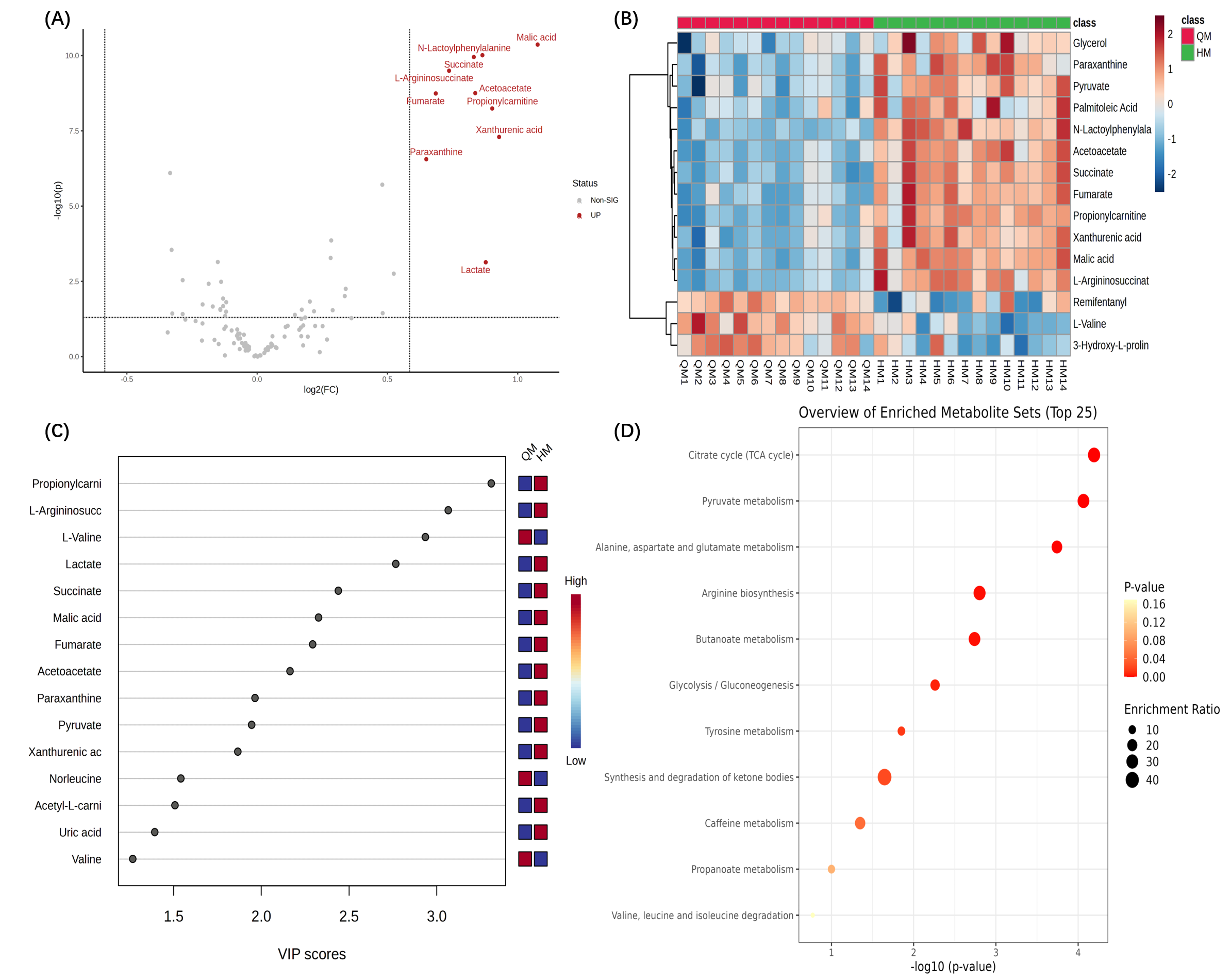

Supplement: Supplementary Figure 2 — Volcano plot (A), Heatmap (B), variable importance in projection (C), and enrichment analysis bubble map (D) of metabolites before and after exercise in M group (n=14). [file Image_2.tif]

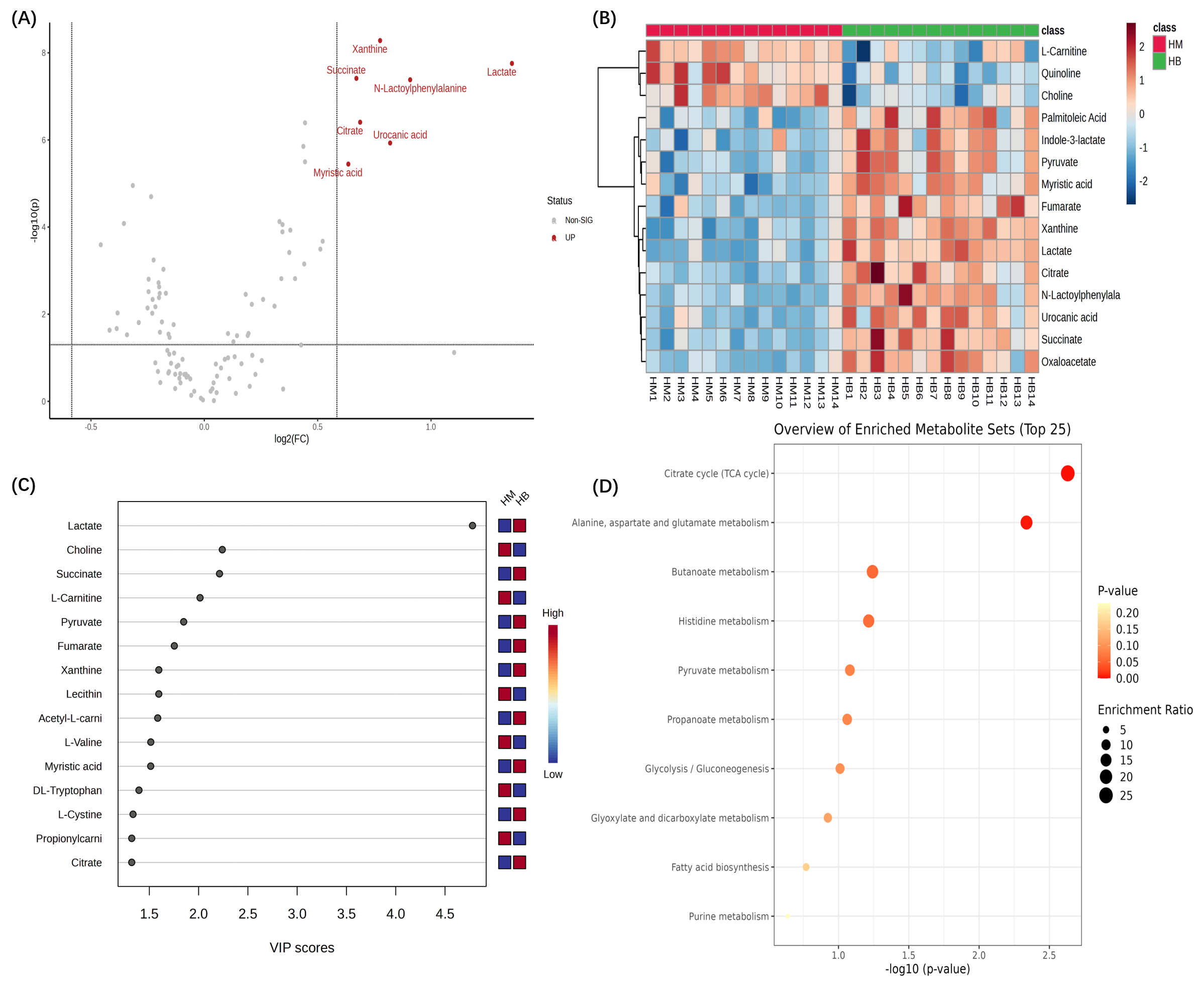

Supplement: Supplementary Figure 3 — Volcano plot (A), Heatmap (B), variable importance in projection (C), and enrichment analysis bubble map (D) of metabolites after exercise between B and M group (n=14). [file Image_3.tif]

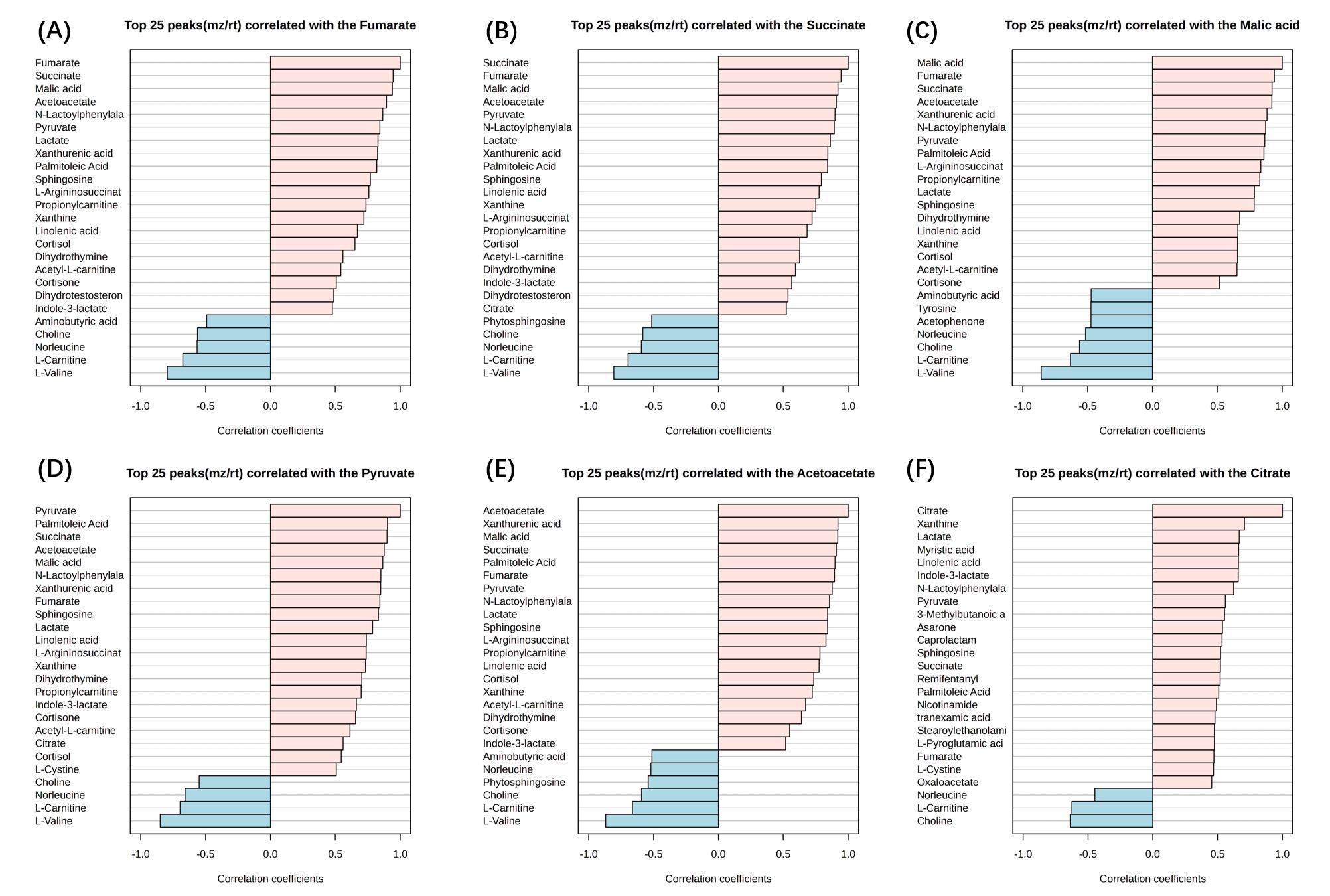

Supplement: Supplementary Figure 4 — Correlation diagram of intermediate metabolites in the citric acid cycle (n=14). [file Image_4.tif]

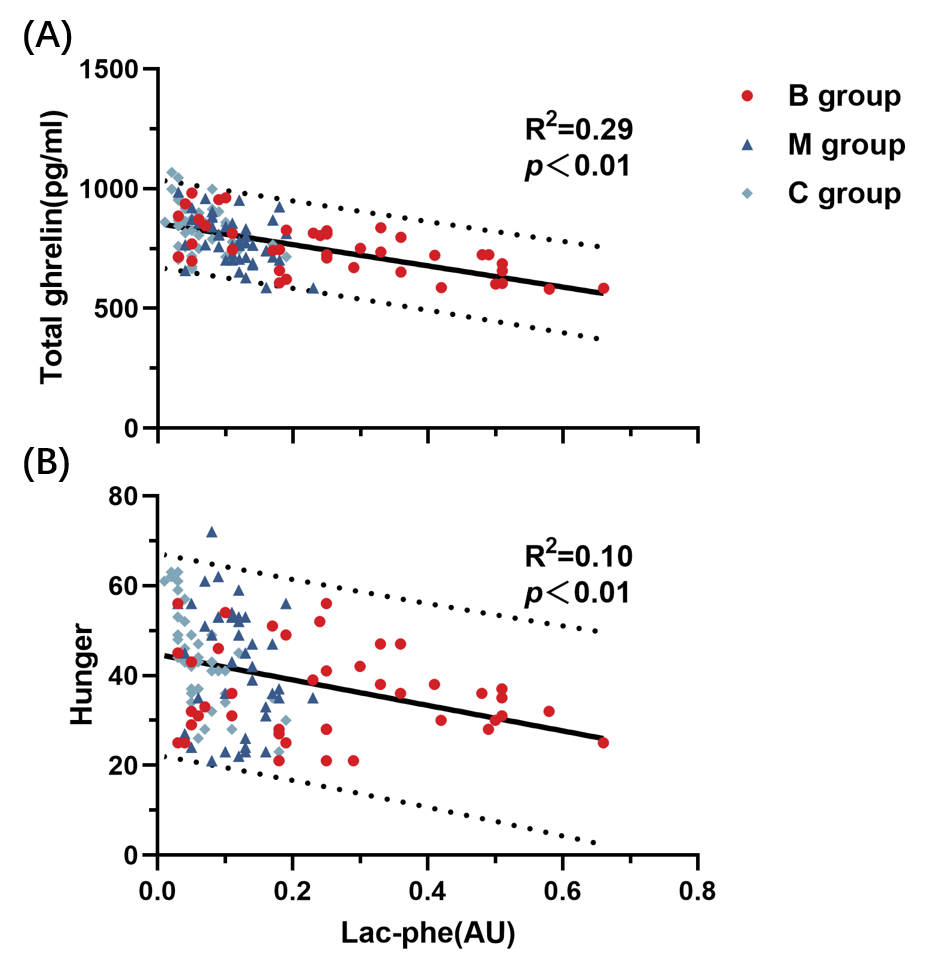

Supplement: Supplementary Figure 5 — Correlation of N-Lactoylphenylalanine with total ghrelin (A) and hunger (B) (n=14). Pearson test was used for correlation analysis. [file Image_5.tif]
