# Supplementary material for: The effect of blood flow restriction exercise on N-lactoylphenylalanine and appetite regulation in obese adults: a cross-design study
Source: Front Endocrinol (Lausanne). 2023 Dec 5;14:1289574. doi: 10.3389/fendo.2023.1289574 (PMC10728722; doi:10.3389/fendo.2023.1289574)
Supplement: Supplementary Table 6 — Food Items Provided in the Ad Libitum Buffet Meal. [file Table_1.docx]

Table S1. Food Items Provided in the Ad Libitum Buffet Meal (n=14)

|  | Energy(kcal/g) | Carbohydrate(g/g) | Protein(g/g) | Lipid(g/g) |
| --- | --- | --- | --- | --- |
| Enregy Proportion(%) | | 55.32 | 8.82 | 35.86 |
| Apple | 0.6900 | 0.1658 | 0.0017 | 0.0017 |
| Banana | 0.8689 | 0.2028 | 0.0122 | 0.0011 |
| Fruit Juice | 0.5380 | 0.1300 | 0.0045 | 0 |
| Whole Yogurt | 0.7441 | 0.0535 | 0.0400 | 0.0412 |
| Potato chips | 5.6667 | 0.4815 | 0.0629 | 0.3889 |
| Chocolate | 4.7800 | 0.6000 | 0.0550 | 0.2400 |
| Toast | 3.8500 | 0.6600 | 0.1550 | 0.0650 |
| Boiled egg | 1.4113 | 0.0060 | 0.1333 | 0.0953 |
| Fruit jelly | 2.4767 | 0.6167 | 0 | 0 |
